# Protective Effect of Argan and Olive Oils against LPS-Induced Oxidative Stress and Inflammation in Mice Livers

**DOI:** 10.3390/ijms18102181

**Published:** 2017-10-19

**Authors:** Soufiane El Kamouni, Riad El Kebbaj, Pierre Andreoletti, Abderrahim El Ktaibi, Issam Rharrassi, Abdelkhalid Essamadi, M’hammed Saïd El Kebbaj, Stéphane Mandard, Norbert Latruffe, Joseph Vamecq, Boubker Nasser, Mustapha Cherkaoui-Malki

**Affiliations:** 1 Laboratoire de Biochimie et Neurosciences, Faculté des Sciences et Techniques, Université Hassan I, BP577, Settat 26000, Morocco; eks.soufiane@gmail.com (S.E.K.); elkebbajriad@gmail.com (R.E.K.); essamadi2002@yahoo.fr (A.E.); 2 Laboratoire des Sciences et Technologies de la Santé, Institut Supérieur des Sciences de la santé Université Hassan I, Settat 26000, Morocco; 3 Laboratoire Bio-PeroxIL EA7270, Université Bourgogne Franche-Comté, UFR SVTE, Dijon 21000, France; pierre.andreoletti@u-bourgogne.fr (P.A.); norbert.latruffe@u-bourgogne.fr (N.L.); 4 Laboratoire d’Anatomie Pathologique Hôpital Militaire Avicenne, Marrakech 40000, Morocco; ab.elktaibi@gmail.com (A.E.K.); rharrassi@yahoo.fr (I.R.); 5 Laboratoire de Biochimie, Faculté des Sciences-Aïn Chock, Université Hassan II-Aïn chock, Casablanca 20000, Morocco; mselkebbaj@yahoo.fr; 6 Lipness Team, INSERM, Research Center UMR866 and LabEx LipSTIC, Université de Bourgogne-Franche Comté, Faculté de Médecine, Dijon 21000, France; stephane.mandard@u-bourgogne.fr; 7 INSERM and HMNO, CBP, CHRU Lille, Lille 59037 and RADEME EA 7364, Faculté de Médecine, Université de Lille 2, Lille 59045, France; joseph.vamecq@inserm.fr

**Keywords:** antioxidant enzymes, argan oil, cytokines, inflammation, lipopolysaccharides, oxidative stress, sepsis

## Abstract

Sepsis causes severe dysregulation of organ functions, via the development of oxidative stress and inflammation. These pathophysiological mechanisms are mimicked in mice injected with bacterial lipopolysaccharide (LPS). Here, protective properties of argan oil against LPS-induced oxidative stress and inflammation are explored in the murine model. Mice received standard chow, supplemented with argan oil (AO) or olive oil (OO) for 25 days, before septic shock was provoked with a single intraperitoneal injection of LPS, 16 hours prior to animal sacrifice. In addition to a rise in oxidative stress and inflammatory markers, injected LPS also caused hepatotoxicity, accompanied by hyperglycemia, hypercholesterolemia and hyperuremia. These LPS-associated toxic effects were blunted by AO pretreatment, as corroborated by normal plasma parameters and cell stress markers (glutathione: GSH) and antioxidant enzymology (catalase, CAT; superoxide dismutase, SOD and glutathione peroxidase, GPx). Hematoxylin–eosin staining revealed that AO can protect against acute liver injury, maintaining a normal status, which is pointed out by absent or reduced LPS-induced hepatic damage markers (i.e., alanine aminotransferase (ALT) and aspartate transaminase (AST)). Our work also indicated that AO displayed anti-inflammatory activity, due to down-regulations of genes encoding pro-inflammatory cytokines Interleukin-6 (IL-6) and Tumor Necrosis Factor-α (TNF-α) and in up-regulations of the expression of anti-inflammatory genes encoding Interleukin-4 (IL-4) and Interleukin-10 (IL-10). OO provided animals with similar, though less extensive, protective changes. Collectively our work adds compelling evidence to the protective mechanisms of AO against LPS-induced liver injury and hence therapeutic potentialities, in regard to the management of human sepsis. Activations of IL-4/Peroxisome Proliferator-Activated Receptors (IL-4/PPARs) signaling and, under LPS, an anti-inflammatory IL-10/Liver X Receptor (IL-10/LXR) route, obviously indicated the high potency and plasticity of the anti-inflammatory properties of argan oil.

## 1. Introduction

Sepsis is characterized by severe organ dysfunctions, requiring intensive care, and is associated with a high mortality rate [1,2]. The host develops an acute syndrome under exposure to endotoxins (lipopolysaccharides, LPS), which are released from bacteria membranes and trigger a potent inflammatory cytokine response, leading to severe impairment of lipid metabolism [3,4,5]. In the liver, the cytokine response is accompanied by a burst of reactive oxygen species (ROS), which causes hepatic dysfunction and, along with proteinases, is concomitant to tissue injury peak [6]. The outcome of this oxidative tissue injury is dependent on the status and adaptive changes in antioxidant pathways [7,8].

Anti-inflammatory and anti-oxidant compounds may be found in traditional herb and plant medicines. Argan oil (AO) is a traditional ingredient in the Moroccan “Amazigh diet”, providing almost 25% of total dietary fat intake to indigenous consumers [9]. We have previously reported that AO has a higher unsaturation index, relative to olive oil (OO) [10]. Hence, OO contains about 70% oleic acid, while AO harbors 45% oleic acid and 35% linoleic acid, indicating that AO is richer in polyunsaturated fatty acids [10]. The earliest clinical studies on argan oil revealed drops in plasma Low Density Lipoprotein-cholesterol (LDL-cholesterol) and lipoperoxides, along with a rise in plasma tochopherol concentration [11]. In addition, the health benefits of this delectable virgin oil have been stressed by several studies documenting its cardiovascular protective potential, in a way that encompasses hypocholesterolemic and hypotriglyceridemic properties, in consumer populations [11,12,13]. More than 20% of patients with sepsis show hepatic metabolism dysregulation and a reduced energy supply for several organs [2]. Dysregulation of lipid metabolism is revealed by reduced serum High Density Lipoprotein (HDL and increased plasma free fatty acids and triglycerides [4]. Metabolic changes are mainly accounted for by increased hepatic triglyceride synthesis and adipocyte lipolysis, which is associated with a reduced level of fatty acid oxidation (FAOx) in several tissues, including heart, kidney, liver and skeletal muscle [4,14,15,16,17]. Mouse models of sepsis use the injection of purified LPS, which induces most pathophysiological changes that develop in human patients [18]. Though the down-regulatory mechanisms by which LPS provokes inflammation and impacts FAOx are well documented, little is known about mechanisms which might protect against these changes. Interestingly, a polyunsaturated fatty acid-rich diet lowers acute inflammation and promotes anti-inflammatory mechanisms in mice [19]. Accordingly, in previous work, we have shown that an argan oil-enriched diet prevents LPS-induced hyperlipidemia through increased liver expression of nuclear Peroxisome Proliferator-Activated Receptor α (PPARα), Estrogen Related Receptor α (ERRα), Peroxisome Proliferator-Activated Receptor Gamma Coactivator-1α, (PGC-1α) and corresponding target genes in FAOx [10,20]. Therefore, AO, as a nutritional supply, might have a preventive action not only on disrupted lipid homeostasis, but also on oxidative stress and inflammatory status.

In this light, AO supplementation has potential to counteract disrupted lipid homeostasis, but also oxidative stress and inflammation caused by LPS, and in this respect obviously deserves additional study, to better delineate its antioxidant/anti-inflammatory properties, and hence, therapeutic potential. Interestingly, numerous studies have focused on the relationship between micronutrients in olive and fish oils and host resistance to infection, particularly when these nutrients are supplemented in the diet to patients at risk of sepsis [21]. Therefore, OO has been suggested as a suitable fat, which may be recommended in clinical nutrition [21]. In this context, the present study was aimed at a more in-depth investigation of the antioxidant and anti-inflammatory properties of argan oil, compared to olive oil, in livers from mice exposed to LPS. Several plasma parameters were studied, along with a histological evaluation of hepatotoxicity. Liver antioxidant capacity was assessed by measurements of antioxidant enzyme activities and reduced glutathione levels. Importantly, pro- and anti- inflammatory gene expressions were concomitantly determined, in order to get a relatively integrated view and decisive breakthrough in our understanding of the molecular basis of AO-driven counteraction of liver sepsis.

## 2. Results

### 2.1. Effect of Argan Oil Pretreatment on LPS-Induced Metabolic Dysregulations of Plasmatic Parameters and Hepatotoxicity

For 25 days, standard chow was given as to two groups (one was later exposed to LPS (LPS group) but not the other (control group)), and was supplemented with either 6% (*w*/*w*) argan oil in two groups (one was later exposed to LPS (AO + LPS group) but not the other (AO group)) or with 6% (*w*/*w*) olive oil in two other groups (one was later exposed to LPS (OO + LPS group) but not the other (OO group)). Table 1 presents the plasmatic parameters in the control and oil-treated groups exposed to LPS. In the two groups treated with either AO or OO alone, no significant modifications were shown for most measured plasmatic parameters. However, regarding glycaemia, contrasting results were obtained for these groups. Indeed, when compared to the control, the AO-group showed a reduction in glycaemia (−22%), while, in contrast, the OO-group revealed an increase (+22%) in glycaemia. Sixteen hours post-LPS injection, LPS-treated mice, when compared to the control, showed a highly significant increase in circulating glucose levels (+59% of glycaemia). Preventive AO or OO supplementation circumvented this effect of LPS treatment. As shown in Table 1, in both the AO + LPS and OO + LPS groups, glycaemia levels were in the normal range.

**Figure 1:**
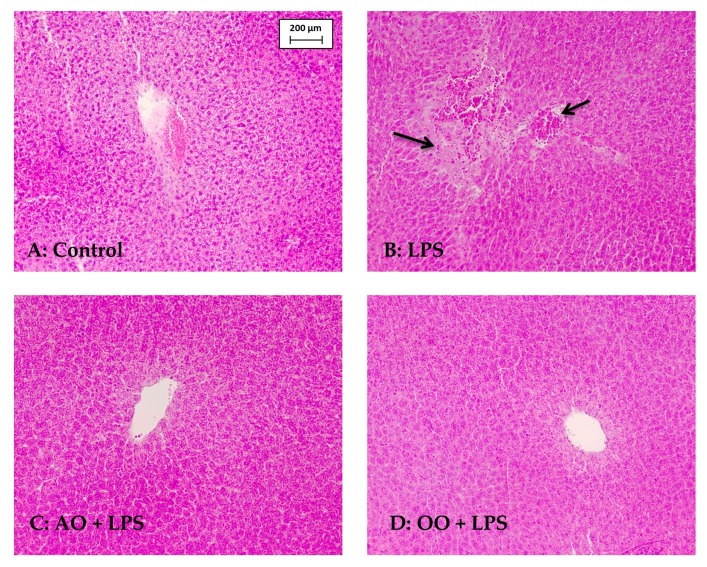
Preventive effect of Argan oil supplementation on LPS (lipopolysaccharide)-induced liver injury. Mice liver sections were stained with hematoxylin–eosin. Control mice (**A**) showed normal liver histologies; LPS group (**B**) showed large foamy lipid-laden macrophages and inflammatory cells (arrows); AO (argan oil) + LPS group (**C**) revealed an interlobular portal vein surrounded by radiating normal hepatocyte plates; (**D**) OO (olive oil) + LPS group showed a normal liver parenchyma surrounding a portal vein (magnification×200).

LPS provoked a moderate rise in blood cholesterol (+18%). Remarkably, AO improved the cholesterol levels in LPS + AO mice and OO pretreatment substantially lowered cholesterol concentrations in LPS + OO mice. On the other hand, uremia was strongly augmented (+265%) by LPS administration, whereas it was significantly reduced in both AO + LPS and OO + LPS mice. However, these values were still significantly higher in control mice. Intriguingly, the AO-enriched diet increased alanine aminotransferase (ALT) by 45%, whereas ALT was only slightly reduced by OO supplementation. However, hematoxylin–eosin staining of liver sections revealed no morphological alterations in either AO or OO group (data not shown). The effect of AO on ALT activity is likely to be related to the phytosterol composition of this oil [22]. Hepatotoxicity of LPS was revealed by significant increases in circulating transaminases activities, including alanine aminotransferase (ALT, +67%) and aspartate transaminase (AST, +21%) activities. Histopathological examination of LPS-treated mice liver sections showed the presence of large foamy lipid-laden macrophages and inflammatory cells (Figure 1), confirming the LPS hepatotoxicity revealed by increased plasmatic AST and ALT activities. The plasmatic protein concentration was also increased (+41%) by LPS treatment, without, however, reaching statistical significance. Remarkably, preventive treatment with AO or OO reversed this LPS hepatotoxic effect, with normal morphology of liver parenchyma in AO + LPS (or OO + LPS). Supplementation with either AO or OO then indicated strong protection by these oils against LPS hepatotoxicity.

### 2.2. Effect of Argan Oil on LPS-Disturbed Antioxidant Activities in Mice Liver Tissues

**Figure 2:**
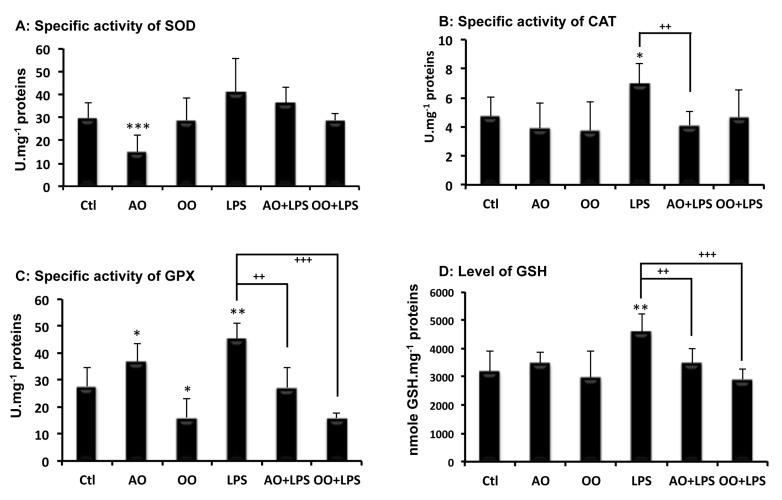
Effects of dietary argan oil and olive oil on mouse liver antioxidant capacities and on their enhancement by LPS. (**A**) Superoxide dismutase (SOD); (**B**) catalase (CAT); (**C**) glutathione peroxidase (GPx) and glutathione (GSH) levels (**D**) Mice received, for 25 days, a standard chow (control: Ctl); a standard chow supplemented with 6% (*w*/*w*) argan oil (AO) or a standard chow supplemented with 6% (*w*/*w*) olive oil (OO). Sixteen hours before euthanasia, separate animal groups, given control, AO and OO-supplemented diets, underwent an intraperitoneal injection of 100 μg LPS (LPS, AO + LPS and OO + LPS groups, respectively). All values are means ± SEM (*n* = 6 per group). Statistical significance of higher mean signal intensity (*** *p* < 0.001, ** *p* < 0.01, * *p* < 0.05) compared to control and (^+++^ *p* < 0.001, ^++^ *p* < 0.01) compared to LPS.

Because LPS-induced hepatotoxicity is known to disturb the intracellular redox balance and to cause oxidative stress [23], the antioxidant capacities of control and treated mice livers were compared. Antioxidant enzyme activities, including superoxide dismutase (SOD), catalase and glutathione peroxidase (GPx), as well as glutathione liver production, were determined. As shown in Figure 2, differential effects of AO or OO supplementation were seen on SOD and GPX activities. While AO decreased SOD activity and increased the GPx activity, on the contrary, OO had no significant effect on SOD but decreased GPx activity. Catalase activity and glutathione levels were neither affected by AO, nor OO, supplementation, in the mouse diet (Figure 2B,D). On the other hand, LPS treatment enhanced liver antioxidant activities; SOD, catalase and GPx activities were increased by +49%, +46% and +58%, respectively (*p* < 0.05 vs. control) (Figure 2A–C), along with a rise in reduced glutathione (+43%) (Figure 2D). Interestingly, the combined treatment of oil with LPS showed a significant decrease in hepatic SOD activity by −48% in AO + LPS mice, and by −49% in OO + LPS mice, respectively, compared to the LPS group (Figure 2A).

Similar results were shown for GPx activity with the combined treatment, with −50% for AO + LPS mice and −57% for OO + LPS mice, respectively (*p* < 0.05 vs. LPS group) (Figure 2C), while hepatic catalase activity and glutathione levels were similar to control values in the combined AO + LPS or OO + LPS treatment groups (Figure 2B,D). These experiments indicated that argan oil, like olive oil, prevents LPS-driven changes in antioxidant activities.

### 2.3. Effect of Argan Oil on LPS-Induced Inflammatory and Anti-Inflammatory Cytokines Disturbed Expression

**Figure 3:**
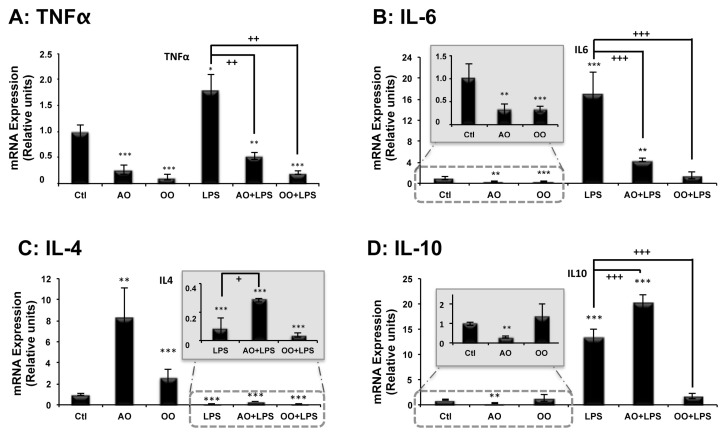
Effects of argan oil and olive oil on LPS-induced disturbances in proinflammatory *Tumor necrosis factor α* (*Tnf-α*) (**A**) and *Interleukin-6* (*Il-6*) (**B**) and anti-inflammatory *Interleukin-4* (*Il-4*) (**C**) and *Interleukin-10* (*Il-10*) (**D**) cytokines’ gene expressions, in mouse liver tissue. Mice received, for 25 days, a standard chow (control: Ctl); a standard chow supplemented with 6% (*w*/*w*) argan oil (AO) or a standard chow supplemented with 6% (*w*/*w*) olive oil (OO). Sixteen hours before euthanasia, separate groups of animals, given control, AO and OO-supplemented diets underwent an intraperitoneal injection of 100 μg LPS (LPS, AO + LPS and OO + LPS groups, respectively). Total RNAs isolated from mouse liver were amplified by RT-qPCR, using specific primers. All real-time PCR reactions were performed in duplicate. All values represent means ± SEM (*n* = 5 per group) and are normalized to the control. Statistical significance of higher mean signal intensity (*** *p* < 0.001, ** *p* < 0.01, * *p* < 0.05) compared to control and (^+++^ *p* < 0.001, ^++^ *p* < 0.01, ^+^ *p* < 0.05) compared to LPS.

LPS species are largely known as proinflammatory compounds [24]. To assess the anti-inflammatory potential of AO, expressions of genes known to encode pro- or anti-inflammatory protein markers were studied by real-time quantitative PCR (qPCR). Remarkably, compared to mice under the control diet, AO supplementation halved hepatic mRNA levels of *Tnf-α* and *Il-6* proinflammatory genes (Figure 3A,B insert) and at the same time, increased by 8-fold, transcription levels of the anti-inflammatory gene, *Il-4*, in the liver (Figure 3C). The other studied anti-inflammatory gene, *Il-10*, was expressed at a low level in the liver, becoming close to the threshold detection level under AO treatment (Figure 3D insert). 

On the other hand, OO supplementation had a similar effect to AO on the mRNA expression of *Tumor necrosis factor-α* (*Tnf-α*) and *Interleukin-6* (*Il-6*) proinflammatory genes, reducing, almost by 3-fold, their expression in OO mice liver relative to the control mice (Figure 3A,B insert). While OO had only a small effect (2-fold) on the transcription level of the anti-inflammatory gene *Interleukin-4* (*Il-4*) and no significant effect on *Interleukin-10* (*Il-10*) gene expression (Figure 3C,D insert). Peritoneal LPS injection induced remarkable hepatic expression of both proinflammatory genes: *Tnf-α* (2-fold) and *Il-6* (16-fold) (Figure 3A,B). Supplementation with AO or OO strongly reduced the LPS-dependent induction of *Tnf-α* and *Il-6* proinflammatory genes, in both AO/LPS and OO + LPS mice groups (Figure 3A,B). Regarding the expression of anti-inflammatory genes, AO supplementation had a specific effect, by inducing both IL-4 and IL-10 mRNA levels in AO + LPS mice, while OO supplementation had no effect (Figure 3C insert and 3D). These results highlighted the unique beneficial effects of AO, in counteracting the LPS-induced proinflammatory effect and boosting hepatic anti-inflammatory gene expression.

## 3. Discussion

The present study affords evidence that an AO-supplemented diet prevents, in mice livers, the dysregulation, by endotoxic LPS shock, of antioxidative capacities and inflammatory status, during endotoxic LPS shock in mice. This preventive effect appears to involve combined coregulation of several pathways, including glucose and lipid metabolism pathways, as well as antioxidative and inflammatory statuses. A six percent dietary supplementation with AO alone had a slight or no effect on plasmatic glycaemia and cholesterol levels, respectively. This is in agreement with previous studies, which have shown that the AO-associated fatty acid supply is compensated by the up-regulation of hepatic expression of PGC-1α, leading to parallel coactivations of the nuclear receptors—PPARα, ERRα and HNF-4α—which govern FAOx metabolism and gluconeogenesis [10,20]. Remarkably, under septic shock, AO (or OO) supplementation counteracted the LPS hyperglycemic effect. This can be explained by the high contentration of argan oil in vitamin E, which is known to lower oxygen free radical generation and to enhance glucose utilization and response to insulin [25]. However, both argan and olive oils contain appreciable amounts of polyphenols, which could exert a hypoglycemic effect. Accordingly, intervention studies in men with a risk of developing metabolic syndrome, with olive leaf polyphenolic compounds (i.e., oleuropein and hydroxytyrosol), have shown significant improvements in insulin sensitivity and pancreatic β-cells secretory capacity after an oral glucose challenge [26]. Similarly, the LPS-induced total cholesterol level was normalized in mice supplemented with AO. This dysregulation of lipid homeostasis has been attributed to the proinflammatory properties of LPS and the cytokines responsible for increases in triglyceride and cholesterol serum levels and hepatic cholesterol synthesis [14,27]. The underlying mechanism has been proposed to involve the sterol regulatory element binding protein (SREBP-1), that governs the transcription of the genes of cholesterol biosynthesis [28], since LPS-stimulated SREBP-1 maturation has been reported in HepG2 cells as well as in mice [29]. AO and OO supplementation decreased plasma cholesterol levels only in the presence of LPS, while being devoid of this effect in the absence of LPS. This may highlight that this AO-related effect on cholesterol levels is dependent on the inflammatory status. It is noteworthy that the cholesterol efflux activities of the ABCA1 and ABCG1 transporters modulate macrophage expression of inflammatory cytokines and chemokines [30]. Accordingly, we previously have shown that AO phytosterols (i.e., schottenol and spinasterol) can modulate gene expressions of the two nuclear receptor Liver X receptor (LXR) isotypes—LXR-α and LXRβ—and their target genes, *Abca1* and *Abcg1* [31]. Interestingly enough, LXR nuclear receptors are considered to be integrators of metabolic and inflammatory signaling [32].

Mice supplemented with OO showed, in the presence of LPS, the strongest decrease (more than under AO) in cholesterol level (57% compared to the control mice). The composition of the OO sterol extract revealed the presence of sitosterol as the main phytosterol (>80%), which is not detected in AO [31]. Interestingly, sitosterol derivatives have been reported to contribute to a decrease in intestinal cholesterol absorption [33], in addition to eliciting an anti-inflammatory effect, by down-regulating several components of the TLR4 pathway [34]. Thus, AO and OO oils each, trigger different mechanisms to induce preventive effects against an LPS-induced rise in the plasma cholesterol level under an inflammatory status.

Here, we show that LPS treatment provoked high uremia, which was partially prevented by AO or OO supplementation. Uremia may occur with acute kidney injury, which is a common manifestation of septic shock [35]. The increase in protein concentration under LPS treatment reveals a protein hypercatabolism, that is often associated with acute kidney injury. Such a situation may consequently cause oxidative stress and lead to increased cytokine production [35]. On the other hand, liver is the main organ targeted by LPS. In our study, LPS-associated hepatotoxicity is indicated by a rise in circulating transaminases (AST and ALT) and attested to by liver cross-section histology. AO or OO supplementation prevents mostly, but not completely, this deleterious effect of LPS due to hepatocyte necrosis, loss of hepatic membrane function and leakage of cellular enzymes into the plasma, including transaminases [36]. Curiously, we also observed a significant increase in serum ALT, only after AO supplementation. To our knowledge, previous epidemiological and experimental studies in human and in animals did not report on hepatotoxicity or a plasma rise in ALT after AO administration [11,37]. Lack of changes in AST activity and in liver histological modifications might argue against an AO hepatotoxic effect. Indeed, it is well known that ALT activity shows daily variations, of 10% to 30%, and its activity becomes higher than AST, because of its longer plasma half-life [38]. However, circulating AST and ALT, though not perfect, are generally considered to be good indicators of liver cytolysis, especially when associated with histological liver studies to corroborate hepatotoxicity, as done in the present work. Nevertheless, serum AST is a less specific marker than ALT in regard to liver function. For instance, in muscle necrosis conditions, serum AST vs ALT, and hence the ratio between AST and ALT serum activity change, preferentially increases [39]. Being aware of the shorter half-life of AST and the tendency of this ratio to evolve towards 1 during muscle necrosis [39], ratios between AST and ALT activity changes in favor of ALT may strengthen the conclusion of a hepatic origin of changes observed in serum AST and ALT. This conclusion is all the more acceptable as the animals were still exposed to, and not at distance to the exposure to, oils and LPS, during the animal sacrifice and blood collection. As shown in Table 1, ALT to AST serum activity changes are in favor of ALT. In this light, the 45% increase induced by argan oil alone in ALT levels may be also concluded to be from hepatic origin. It might be concluded to result from liver cytolysis. However, argan oil supplementation is not associated with histological signs of hepatotoxicity and even cancels those induced by LPS. In this context, and in view of a previous work highlighting the positive effects of argan oil alone in up-regulating hepatic gluconeogenesis genes (notably encoding phosphoenol–pyruvate carboxykinase (PEPCK) and glucose-6-phosphatase) [20], an elegant explanation might be found in increased liver production and a consequent rise in circulating ALT. Indeed, a liver overproduction of ALT may further contribute to the enhanced gluconeogenic effect induced by argan oil, since ALT is the first liver enzyme involved in the gluconeogenicity of alanine (largely released by extra-hepatic tissues in the circulation during starvation in the scope of the Cori’s cycle [40,41,42]. ALT catalyzes the conversion of alanine to pyruvate, which is metabolized by pyruvate carboxylase to oxaloacetate, itself further handled by PEPCK and gluconeogenesis.

LPS dysregulation of antioxidative capacities was shown by increased activities of antioxidant enzymes, including SOD, catalase and GPx, and the stress marker glutathione. Excessive production of reactive oxygen species (i.e., superoxide, hydrogen peroxide and hydroxyl radicals) is well associated with liver injury during endotoxic shock. Numerous studies have reported that LPS treatment increases antioxidant enzyme activities [20,43] and therefore, in this particular context, enhanced antioxidant defense capacities should be viewed as a response paralleling antioxidant injury, rather than as a cause of tolerance to oxidative stress. Two main transcription factors play key roles in the regulation of the expression of genes encoding antioxidant enzymes. Forkhead box O (FOXO) transcription factors promote their antioxidant effects through the regulation of SOD and catalase. However, FOXO also plays a role in the regulation of GSH-mediated detoxification, through the transcriptional upregulation of GPx1 [44]. On the other hand, an increase in cytosolic oxidative stress leads to the dissociation of nuclear factor erythroid 2-related factor 2 (Nrf2) from the Nrf2–Keap1 complex and its translocation to the nucleus for the activation of the Nrf2/antioxidant response element. Nrf2 controls the transactivation of genes that code for the proteins necessary for glutathione synthesis and electrophile detoxification, including ROS-detoxifying enzymes, such as GPX2 and several glutathione S-transferases [44]. In this context, AO and OO supplementation improved antioxidant status, as attested to by the preservation of normal levels in the AO + LPS or OO + LPS groups. The antioxidant protective effect of AO is related to its high content in polyphenols (particularly ferulic acid), tocopherols, polyunsaturated and unsaturated fatty acids, and β-carotene. Most of these compounds are capable of blunting oxidative injuries through free radical scavenging mechanisms [45].

Diets supplemented with AO alone (or OO as well) exhibited sharp preventive anti-inflammatory properties, decreasing pro-inflammatory cytokines *Tnf-α* and *Il-6* gene expression levels and in the presence of LPS, this AO-preventive anti-inflammatory effect was highly significant. In the liver, LPS activate Kupffer cells (as hepatic resident macrophages), which can release cytotoxic agents, such as TNF-α and IL-6, through the activation of the nuclear factor NF-κB signaling pathway [46]. In Kupffer cells, the upregulation of TNF-α further contributes, in an autocrine manner, to increased IL-6 expression, which is regulated by the LPS/Myeloid differentiation primary response 88 (LPS/MyD88) pathway [47]. The preventive effect of AO against the activation of the pro-inflammatory genes, *Tnf-α* and *Il-6*, in Kupffer cells, could be related to the activation of hepatic PPARα by AO polyunsaturated fatty acids. Indeed, activation of hepatic PPARα expression by AO (or OO), leads to inhibition of the Nuclear Factor-κB (NF-κB) signaling pathway. This inhibition involves induction of the IκBα factor, which sequesters NF-κB in the cytosol and subsequently abrogates the nuclear NF-κB-dependent activation of *Tnf-α* and *Il-6* gene expressions [48,49].

The strong hepatic activation of *Il-4* gene expression illustrates the benefit related to AO supplementation. Whereas IL-4 has striking inhibitory effects on the expression of proinflammatory cytokines [50], IL-4 signaling was also reported to regulate the metabolic switch between glucose and fatty acid oxidation [51]. Previous studies have shown that cooperation between IL-4/STAT6 signaling, PPARs and PGC-1 governs the alternative macrophage reprograming for oxidative metabolism through the up-regulation of fatty acid oxidation and mitochondrial biogenesis [52]. IL-10 is also an important immunoregulatory factor that significantly inhibits monocytes/macrophage-derived proinflammatory cytokines, including TNF-α and IL-6 [53]. Furthermore, the activation of LPS-induced NF-κB can be achieved by IL-10 in macrophages and pre-B cells [54]. Accordingly, macrophage deactivation is promoted by PPARδ and LXRs activation, by increasing IL-10 release and restraining IL-12 and TNF-α delivery, leading to the suppression of inflammation [52].

Regarding present and previous studies on AO effects, we revealed that AO regulates glucose and FAOx pathways through the activation of the nuclear receptors—PPARα, ERRα and HNF-4α—and their coactivator, PGC-1α, in addition to the LXR-dependent modulation of cholesterol metabolism [20,31]. Taking into account the key roles of PPARα and LXRs as integrators of metabolic and inflammatory signaling, we can now further support that AO supplementation activates IL-4/PPARs signaling, and under LPS, amplifies the anti-inflammatory response in the liver through the IL-10/LXR route. This novel advance originally highlights the high potency and plasticity of the anti-inflammatory properties of argan oil.

## 4. Material and Methods

### 4.1. Argan Oil Treatment

Swiss OF1 mice (12 to 16 weeks-old) were obtained from IFFA CREDO (Casablanca, Morocco). They were acclimatized in the laboratory for 10 days at 22 ± 2 °C with standard chow and water given ad libitum. Animal studies were conducted in accordance with the protocols of Animal Use and Care of the University of Hassan 1st, Settat, Morocco. The virgin argan oil used in this work was obtained from the Aklim area in the northeast of Morocco. Eight groups of mice (6 mice/group) received, for 25 days: a standard chow (2 groups, control); a standard chow supplemented with 6% (*w*/*w*) argan oil (2 groups, AO) or a standard chow supplemented with 6% (*w*/*w*) olive oil (2 groups, OO). Oils were included in the diets by direct mixing with the standard animal chow. Sixteen hours before euthanasia and during the fed state, one group from the control (+LPS), AO (AO + LPS) and OO (OO + LPS) groups received (5 mg/kg) intraperitoneal injections of 100 μg of *Escherichia coli* 0111:B4 LPS (Sigma, Saint-Quentin-Fallavier, France), resuspended in phosphate-buffered saline (PBS) or an equal volume of PBS alone. The mouse blood was collected in untreated test tubes and incubated undisturbed, without anticoagulant, at room temperature, for 20 min. The tubes were then centrifuged at 3000× *g* for 15 min at 4 °C and the resulting plasma was kept at −80 °C. Liver tissues were immediately frozen in an ethanol-dry ice bath and stored at −80 °C.

### 4.2. Preparation of Homogenates

A 10% (*w*/*v*) homogenate was prepared in a potassium phosphate buffer 0.05 M, pH 7.4, using potter Elvehjm homogenizer (Dominique Dutscher, Issy-les-Moulineaux, France). The homogenate was centrifuged for 10 min at 3000× *g* at 4 °C and the supernatant aliquots were saved and stored at −20 °C. The homogenate protein content was measured, according to the method described by Lowry et al. [55], using bovine serum albumin as a standard.

### 4.3. Plasmatic Parameters Estimation

Measurements of the plasmatic AST and ALT activities, and concentrations of glucose, cholesterol, proteins and urea, were determined using the Cobas e411 analyzer (Roche Diagnostics, Mannheim, Germany), according to the controls and calibration of the Cobas e411 analyzer (Roche Diagnostics, Mannheim, Germany).

### 4.4. Reduced Glutathione Level

Reduced glutathione (GSH) levels in liver homogenates were determined according to Ellman [56]. Briefly, trichloroacetic acid solution was mixed with homogenates. After centrifugation for 10 min at 12,000× *g*, the supernatant was collected and mixed with a phosphate buffer (50 mM, pH 8) and 6 mM 5,5-dithiobis (2-nitrobenzoic acid (DTNB)). The yellow-colored solution was immediately read at 412 nm, using an Ultra-violet-visible spectrophotometer. Results were calculated based on a standard glutathione curve and expressed as μmol of GSH/mg of protein.

### 4.5. Catalase Activity

Catalase activity was assessed by adding 0.05 mL of the extract in a quartz cuvette (3 mL), containing 1 mL of 0.019 M hydrogen peroxide and 1.95 mL of potassium phosphate buffer (0.05 M, pH 7.4) [57]. The activity was determined by monitoring the decreasing rate of absorbance at 240 nm.

### 4.6. Superoxide Dismutase Activity

The activity of superoxide dismutase was assayed as described by Beyer and Fridovich [58]. A 3mL mixture reaction, containing 50 mM phosphate buffer, 0.025% triton x-100, 0.1 mM EDTA (pH 8), 12 mM l-Méthionine, 75 mM nitro blue tetrazolium chloride NBT, and enzyme aliquot and 2 μM riboflavin. The activity was measured at 560 nm.

### 4.7. Glutathione Peroxidase Activity

The GPx activity was measured according to the method of Flohé and Günzler [59]. For the enzyme reaction, 0.3 mL of the extract was placed into a tube and mixed with 0.1 M potassium phosphate buffer (pH 7.0), 2 mM GSH (reduced glutathione), 0.5 mM EDTA, and 1 mM sodium Azide and 1 mM H_2_O_2_. After incubation at 37 °C for 15 min, the reaction was stopped by adding 0.5 mL of TCA (5%) and then centrifuged at 1500× *g* for 5 min. 0.1 mL of supernatant was added to 0.2 mL of phosphate and 0.7 mL of DTNB buffer (0.4mg/mL). Reading of the optical density was carried out at 420 nm.

### 4.8. Histological Analysis

For light microscopy, pieces of liver were fixed in 10% formalin, embedded in paraffin, and 5-μm-thick sections were stained with hematoxylin and eosin, as described by Vluggens et al. [60].

### 4.9. Quantitative PCR Analysis

Total RNA from liver was extracted using the RNeasy Mini kit (Qiagen, Les Ulis, France) following the manufacturer’s instructions. cDNA was generated by reverse transcription, using Moloney Murine Leukemia Virus Reverse Transcriptase (Promega, Charbonnières-les-Bains, France), according to the manufacturer’s protocol, and analyzed by quantitative PCR, using the GoTaq^®^ qPCR Master Mix (Promega, Charbonnières-les-Bains, France), and a StepOnePlus Real-Time PCR System (Applied Biosystem, Villebon Sur Yvette, France). The primer sequences were chosen using Beacon Designer Software (Bio-Rad, Marnes-la-Coquette, France). Oligonucleotide sequences are detailed in Supplementary Table 1. PCR reactions were carried out in duplicate in a final volume of 12.5 μL, containing 6.25 μL of MESA Green qPCR Mastermix (Eurogentec, Angers, France), 2.5 μL of cDNA and forward and reverse primers at 300 nm. The PCR enzyme (Taq DNA polymerase, Promega, Charbonnières-les-Bains, France) was heat-activated at 95 °C for 10 min, and the DNA was amplified for 40 cycles at 95 °C for 15 s, 60 °C for 30 s, and 72 °C for 30 s, followed by a melting curve analysis to control the absence of nonspecific products. For each transcript, the amplification efficiency was determined by the slope of the standard curve generated from two-fold serial dilutions of cDNA. Gene expression was quantified using cycle to threshold (Ct) values and normalized by the reference gene, 36B4, encoding the acidic ribosomal phosphoprotein P0. To this end, the quantitative gene expression was determined according to 2^−ΔΔ*C*t^ with Δ*C*_t_ = (*C*_t_ of the gene studied)–(*C*_t_ of the 36B4 gene).

### 4.10. Statistical Data Analysis

Statistical analyses to compare two experimental groups were performed with an unpaired, two-tailed, Student-*t* test (Excel software) for calculating the probability values and data were considered statistically different at a *p*-value of 0.05 or less.

## Figures and Tables

**Table 1:** Protective effects of argan oil (AO) and olive oil (OO) against LPS (lipopolysaccharide)-induced dysregulation, in regard to plasmatic biochemical parameters.

Plasma Parameters	Control	LPS	AO	AO + LPS	OO	OO + LPS
**Glycaemia (mg/dL)**	2.91 ± 0.08	4.63 ± 0.6 **	2.28 ± 0.21 *	3.7 ± 0.5 **	3.56 ± 0.23 *	3.48 ± 0.39 ^##^
	100%	159% ^a^	78.3% ^a^	80% ^b^	122% ^a^	75.2% ^b^
**Cholesterol (mg/dL)**	1.97 ± 0.11	2.33 ± 0.28*	1.96 ± 0.13	2.07 ± 0.06	1.66 ± 0.15 *	1.02 ± 0.03 ***^##^
	100%	118% ^a^	99.5% ^a^	88.8% ^b^	84.3% ^a^	43.8% ^b^
**Proteins (mg/dL)**	48.17 ± 7.89	68.15 ± 12.8 *	42.60 ± 2.67	45.75 ± 0.9 ^#^	51.83 ± 5.8	38.80 ± 3.39 ^#^
	100%	141% ^a^	88.4% ^a^	67.1% ^b^	107.6% ^a^	56.9% ^b^
**Uremia (mmol/dL)**	6.35 ± 0.07	23.17±2.29 ***	6.6 ± 0.36	19.18 ±1.18 ***^#^	4.80 ± 0.99	16.95 ± 0.07 ***^##^
	100%	365% ^a^	104% ^a^	82.8% ^b^	75.6% ^a^	73.15% ^b^
**AST (U/mL)**	395.5 ± 7.78	480.8 ± 11.6 ***	387.1 ± 0.71	295 ± 8.49 ***^###^	329.95 ± 13.79 **	292 ± 16.97 ***^###^
	100%	121.6% ^a^	97.9% ^a^	61.4% ^b^	83.4% ^a^	60.7% ^b^
**ALT (U/mL)**	43.45 ± 2.19	72.9 ± 1.56 ***	63.03 ± 1.78 ***	47.3 ± 5.80 ^###^	37.9 ± 3.96	48.05 ± 10.11 ^##^
	100%	167.8% ^a^	145% ^a^	64.9% ^b^	87.2% ^a^	65.9% ^b^
ALT to AST % increase ratio	1	1.38	1.48	1.05	1.05	1.09

All values are means ± SEM (*n* = 6/group). Statistical significance of higher mean signal intensity (*** *p* < 0.001, ** *p* < 0.01, * *p* < 0.05) compared to control and (^###^ *p* < 0.001, ^##^ *p* < 0.01, ^#^ *p* < 0.05) compared to LPS. Fold change in percent was calculated for each group by dividing the mean of the LPS group, AO group or OO group per mean of the control group (^a^) and by dividing the mean of the AO + LPS group or OO + LPS group per mean of the LPS group (^b^). AST: aspartate transaminase; ALT: alanine aminotransferase.

## Supplementary Materials

Supplementary materials can be found at www.mdpi.com/1422-0067/18/10/2181/s1.
