# Supplementary material for: Protective Effect of Argan and Olive Oils against LPS-Induced Oxidative Stress and Inflammation in Mice Livers
Source: Int J Mol Sci. 2017 Oct 19;18(10):2181. doi: 10.3390/ijms18102181 (PMC5666862; doi:10.3390/ijms18102181)
Supplement: Supplementary file 1 [file ijms-18-02181-s001.pdf]

Supplementary Table 1. Primers sequences used for the quantitative real-time PCR experiement.

Gene name	Primers
- 36B4 *forward:*	5'-ATCTGCTTGGAGCCCACAT-3'
- 36B4 *reverse:*	5'-GCGACCTGGAAGTCCAACTA-3'
- IL-6 *forward:*	5'-TCTATACCACTTCACAAGTCGGA-3'
- IL-6 *reverse:*	5'-GAATTGCCATTGCACAACTCTTT-3'
- IL-4 *forward:*	5'-CCATATCCACGGATGCGACAA-3'
- IL-4 *reverse:*	5'-CCTCGTTCAAAATGCCGATGAT-3'
- IL-10 *forward:*	5'-GCTGGACAACATACTGCTAACC-3'
- IL-10 *reverse:*	5'-CCCAAGTAACCCTTAAAGTCCTG-3'
- TNF-α *forward:*	5'-CCCTCACACTCAGATCATCTTCT-3'
- TNF-α *reverse:*	5'-GCTACGACGTGGGCTACAG-3'
